# Pharmacokinetics and bioequivalence of two cyclosporine oral solution formulations in cats

**DOI:** 10.3389/fvets.2022.940472

**Published:** 2022-08-10

**Authors:** Yuxin Yang, Jingyuan Kong, Yu Liu, Qinyao Wu, Yuying Cao, Jicheng Qiu, Lu Zhang, Xiaohui Gong, Fuhua Zhao, Xingyuan Cao, Jianzhong Wang

**Affiliations:** ^1^Department of Veterinary Pharmacology and Toxicology, College of Veterinary Medicine, China Agricultural University, Beijing, China; ^2^China Institute of Veterinary Drug Control, Beijing, China; ^3^Key Laboratory of Detection for Veterinary Drug Residues and Illegal Additives, Ministry of Agriculture and Rural Affairs of the People's Republic of China, Beijing, China; ^4^Shanxi Key Laboratory for Modernization of TCVM, College of Veterinary Medicine, Shanxi Agricultural University, Jinzhong, China

**Keywords:** pharmacokinetics, bioequivalence, cyclosporine oral solution, cat, four-cycle

## Abstract

The pharmacokinetic profiles and bioequivalence of two cyclosporine oral solutions were investigated in cats. Twenty-four cats were randomly allocated to two equally sized treatment groups in a randomized four-cycle, and dual-sequence cross-over design. Test and reference articles were orally administered in a single dose of 7 mg/kg Bodyweight. Serial blood samples were collected, and blood cyclosporine concentration was determined by ultra-performance liquid chromatography-mass spectrometry (UPLC-MS/MS). No significant differences were present in the major pharmacokinetic parameters (C_max_, AUC_0−last_,) between the two formulations. The blood profiles of cyclosporine following the administration of both formulations were similar. The findings of the study suggested that the two articles were bioequivalent for cyclosporine oral solution.

## Introduction

Atopic dermatitis (AD) is a T cell-dependent common, chronic, relapsing inflammatory skin disease; however, therapeutic options for patients with the moderate-to-severe disease are limited (1–3). Moreover, affected individuals typically have pruritic erythematous lesions, as well as secondary skin lesions in curved and rubbed areas (4, 5). Cyclosporine A (CyA) is a calcineurin inhibitor. It is a powerful immunosuppressant drug that acts by inhibiting the proliferation of T-lymphocytes (6, 7). CyA's direct effect is *via* inhibition of calcineurin and exhibits an immunosuppressive effect by inhibiting cytokines, which are secreted by T lymphocytes (8–10). Cyclosporine is lipophilic, distributes widely, and is stored in the skin and adipose tissue. Its concentration in the epidermis and dermis is about 10 feet higher than in blood (11–13). Cyclosporine was proven efficacious in the treatment of feline hypersensitivity dermatitis (14). Cyclosporine oral solution was approved by the US Food and Drug Administration as ATOPICA for Cats^®^ (Cyclosporine oral solution, USP) for the control of feline hypersensitivity dermatitis in cats.

CyA is a narrow therapeutic index drug, and in individuals, there are differences in pharmacokinetics and bioavailability of cyclosporine in large parts (15, 16). Due to the extreme variability in absorption and metabolism, monitoring the concentrations of CyA in the blood has been recommended to reduce the occurrence of adverse drug events and maximize the treatment effect (17). CyA concentration should be evaluated in the whole blood rather than just plasma because the drug concentrates within blood cells (18). Ideally, testing should be carried out after 2 weeks of treatment and, where available, high-performance liquid chromatography is a better method than immunoassay for evaluating CyA whole blood concentrations (19).

In recent years, very few studies have been published specifically addressing the pharmacokinetics of CyA in feline species. This study was conducted to compare the pharmacokinetic profiles of generic cyclosporine manufactured by Shanghai Hanwei Biomedical Technology Co., Ltd. (Shanghai, China) with Atopica™ (Elanco Australasia Pty Ltd.) to evaluate their bioequivalence and, consequently, the possibility of substitution between the two drugs in cats.

## Materials and methods

### Materials

The cyclosporine oral solution (Shanghai Hanwei Biomedical Technology Co., Ltd, 30 mL/bottle, 100 mg/mL) was the Test Product, while Atopica™ was used as the reference formulation (Elanco Australasia Pty Ltd., 17 ml/bottle, 100 mg/ml). Cyclosporine Standard product was provided from Shanghai Hanwei Biomedical Technology Co., Ltd. (purity: ≥99%).

### Study design

Twenty-four domesticated shorthair cats (aged 2–3 years and weighing between 3 and 4.5 kg, provided by the Experimental Animal Center of China Agricultural University) were enrolled in this study. Cats fasted for 16 h before and 8 h following drug administration. Before the initiation of the study, all procedures were reviewed and approved by the Institutional Animal Care and Use Committee of the China Agricultural University (No. 13303-21-E-001).

The study was conducted in a single dose, a four-way fully replicated, and crossover design. A 2-week washout period was scheduled between each phase. Twenty-four cats were randomly blocked into two groups. Cats were monitored for other potential adverse effects during the study. The oral solution was administered *via* a dosing syringe to the back of the tongue in four phases. In brief, cyclosporine PK data were collected as follows:

- In phases 1 and 3, the 12 cats in Reference-Test- Reference-Test (RTRT) groups were administrated with 7 mg/kg Bodyweight (BW) reference formulation, while administrated with 7 mg/kg BW test formulation in Test-Reference-Test-Reference (TRTR) groups.
- In phases 2 and 4, cats in the RTRT group were administrated with a 7 mg/kg BW test formulation, and the TRTR group was administrated 7 mg/kg BW reference formulation.

Blood samples of about 0.8 mL were collected *via* the brachial cephalic vein at 0, 0.25, 0.5, 0.75, 1, 2, 3, 4, 6, 8, 12, 24, 36, 48, and 72 h after dosing. Whole blood samples were immediately placed in an anticoagulation blood collection tube and stored at −20°C until analysis.

### Drug analysis

Cyclosporine concentrations in plasma samples were measured using a validated UPLC-MS/MS analytic method as previously described (20). In brief, 200 μl of blood was mixed with 20 μl methanol and 400 μl acetonitrile: methanol (1:1), vibrated for 2 min. After centrifugation at 12,000 rpm at 4°C for 20 min, 300 μl of the supernatant was centrifugated at 12,000 rpm at 4°C for 10 min. The supernatant was analyzed *via* UPLC-MS/MS (Waters Acquity UPLC and Water Quattro Premier, Waters Co, USA). The mobile phase consisted of 2-mm ammonium acetate 0.1% formic acid (solvent A) and methanol containing 2-mm ammonium 0.1% formic acid (solvent B) with a flow rate of 0.30 ml/min (The mobile phase ratio is shown in Table 1). The lower limit of quantification (LLOQ) was 10 ng/ml. Both inter- and intra-assay coefficients of variation were <15%. The mean recoveries ranged from 93.39 to 110.72%. Calibration curves showed satisfactory linearity through a concentration range of 10–2,000 ng/ml (*r*^2^ > 0.99) (21).

**Table 1:** Gradient elution conditions of ultra-high performance liquid chromatography (UPLC).

**Time (min)**	**Solvent A (%)**	**Solvent B (%)**
0	80	20
0.8	80	20
2.5	2	98
4.0	2	98
4.2	80	20
6	80	20

### Data analysis

CVM advocates the use of 90% confidence intervals (CI), as the best available method for evaluating bioequivalence study data. The pivotal variables for bioequivalence are AUC_last_, AUC_INF_obs,_, and C_max_. Mixed model analysis was used to estimate upper and lower bounds for the two pivotal bioequivalence parameters, AUC_last_, AUC_INF_obs,_, and C_max_. The recommended BE limit is 80–125% (22, 23). In this study, blood pharmacokinetic parameters were calculated using the non-compartmental analysis model 200 (intravenous or extravascular dosing, linear/log trapezoidal method) in the WinNonlin™ software (version 8.1; Certara USA) and WinNonlin 8.1 was used for bioequivalence analysis. Analysis of variance (ANOVA) was used to calculate a 90% CI for the ratio of the two treatments (24, 25).

## Results

**Figure 1:**
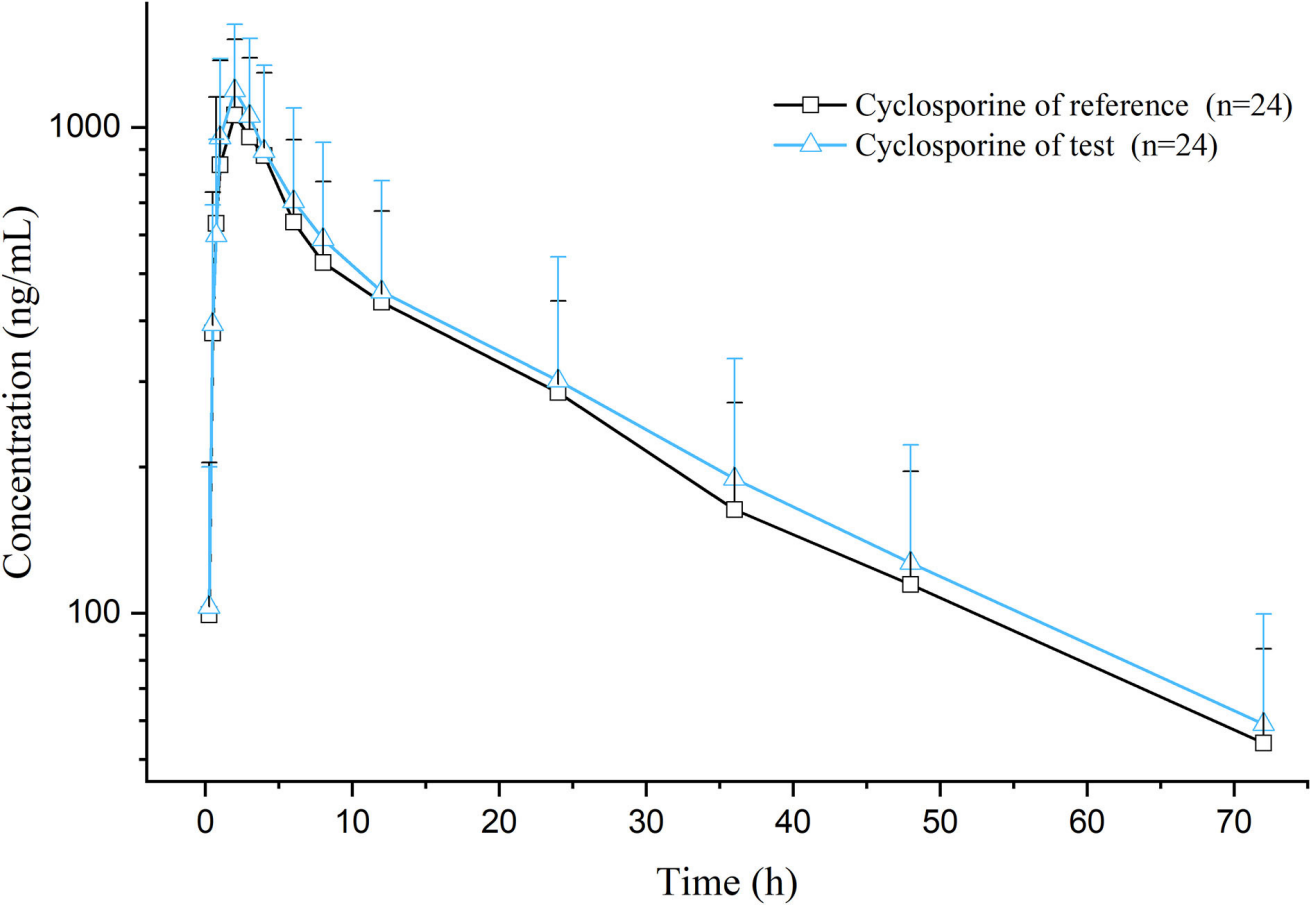
Semilogarithmic plot of the cyclosporine plasma concentrations–time curves.

After a single oral dose of 7 mg/kg BW of cyclosporine reference and test formulations in cats (after administration and throughout the experimental process, the cats were in good condition, and no adverse reactions occurred), the average blood concentration-time curve corresponding to the test and the reference formulations measured is presented in Figure 1. The pharmacokinetic parameters were calculated using non-compartmental analysis, and the results are presented in Table 2. The geometric mean ratios of the test formulation/reference formulation C_max_, AUC_last_, AUC_INF_obs,_, and their 90% CI are presented in Table 3, which indicates that the test and reference formulations are bioequivalent.

**Table 2:** Pharmacokinetic variables obtained for two formulations of cyclosporine in cats (*n* = 24) after a single dose of 7 mg/kg orally.

**Parameters**	**Units**	**Reference**	**Test**
		**formulation**	**formulation**
*T_*max*_*	h	2.22 ± 0.77	2.21 ± 0.92
*C_*max*_*	ng/mL	1244.96 ± 489.63	1319.57 ± 403.39
*AUC_*last*_*	h·ng/mL	18556.25 ± 8133.34	20153.16 ± 11304.13
*AUC_*INF*_*obs*_*	h·ng/mL	20506.12 ± 8711.34	22355.21 ± 12246.25

Pharmacokinetic parameters were calculated using Non-Compartmental Analysis Model 200 in WinNonlin™ software; T_*max*_, the time after the initial injection to when C_*max*_ occurs; C_*max*_, maximum plasma concentration; AUC_*last*_, area under the concentration versus time curve from 0 to the last point; AUC_*INF_obs*_, area under the concentration vs. time curve from 0 to infinity.

**Table 3:** BE analysis of cyclosporine test and reference formulations.

**Parameters**	**Ratio_%Ref_**	**90% CI range**	
		**Lower**	**Upper**
		**limit (%)**	**limit (%)**
Ln (C_max_)	107.23	93.39	123.12
Ln (AUC_last_)	100.44	82.73	121.94
Ln (AUC_INF_obs_)	100.41	83.05	121.41

## Discussion

After oral administration of the test and the reference formulations, the pharmacokinetic parameters of the test and the reference formulations: T_max_, C_max_, AUC_0−t_, and T_1/2_ were not significantly different. After bioequivalence analysis of AUC_0−t_, AUC_0−∞_, and C_max_, the CI 90 ranges of the test formulation compared with the reference formulation was between 80 and 125%. The test formulation is bioequivalent to the reference formulation.

When administered to cats, the plasma kinetic profile of CyA was apparently different from that of other domestic animals, and different formulations had different results. In this study, T_max_ was achieved after 2.21 ± 0.92 h, which is shorter than what was reported for beagle dogs (6 ± 0.00) that were administered at 75-mg sustained-release pellets (26), rats (4 ± 2.40) after being administered with 37.8 mg/kg BW (27). Also, it is slightly longer than what was reported for dogs (1.40 ± 0.30 h) that were administered with a capsule of 5 mg/kg BW (28), and rabbits (1.75 ± 0.76 h) after being administered with an oral solution of 10 mg/kg BW (29). Our results showed that administration of CyA at 7 mg/kg in cats had a relatively rapid absorption and distribution in contrast to some studies. When CyA was administered orally (75 mg sustained-release pellets) in dogs, the AUC_0−24_ was 3,205 ± 149.55 ng·h/ml (26). The AUC_0−24_ and AUC_0−∞_ of CyA at 10 mg/kg BW after oral administration to rabbits were 2,057.80 ± 778.60 ng·h/ml and 3,492.90 ± 1,449.70 ng·h/ml, respectively (29). The AUC_0−∞_ value following oral administration at 5 mg/kg BW in dogs was 3,997 ± 1,108 ng·h/ml (28). The AUC_0−t_ and AUC_0−∞_ values in this study were 20,153.16 ± 11,304.13 ng·h/ml and 22,355.21 ± 12,246.25 ng·h/ml, respectively. These results showed that this study had a higher AUC than others, the relative accumulation of the drug in the blood is greater, and higher availability of CyA was reflected in cats than in other animals. In this experiment, the C_max_ of the test formulation was 1,319.57 ± 403.39 ng/ml. In research about 75 mg sustained-release pellets of CyA in beagle dogs (26), the C_max_ was 268.22 ± 15.99 ng/ml. Pharmacokinetics and efficacy of canine atopic dermatitis study of CyA in dogs, the C_max_ of CyA capsule (5 mg/kg) was 577 ± 158 ng/mL (28). The C_max_ of CyA at 10 mg/kg BW after oral administration to rabbits was 244.67 ± 115.87 ng/ml (29). Compared with the other study, the C_max_ of this experiment is higher, indicating that its concentration in the blood is higher and the drug has a stronger effect.

## Conclusion

In this experiment, the results of pharmacokinetic process analysis showed that the test formulation of CyA oral liquid had the characteristics of fast absorption and slow elimination in cats. The relative bioavailability of the test formulation of CyA oral solution was 108.61%, and the test formulation of CyA and the reference formulation were bioequivalent.

## Data availability statement

The original contributions presented in the study are included in the article/Supplementary material, further inquiries can be directed to the corresponding author/s.

## Ethics statement

The animal study was reviewed and approved by Institutional Animal Care and Use Committee of the China Agricultural University (No13303-21-E-001).

## Author contributions

XC and JW contribute to revising it critically for important intellectual content and approved the version to be published. YY, JK, FZ, YL, QW, YC, JQ, LZ, and XG have participated sufficiently in the work to take public responsibility for appropriate portions of the content and made a substantial contribution to the concept and design, acquisition of data or analysis, and interpretation of data. YY drafted the article. All authors contributed to the article and approved the submitted version.

## Funding

This work was supported by Central Funds Guiding the Local Science and Technology Development In Shanxi Province [Grant Number YDZJSX2021A034], Fund Program for the Scientific Activities of Selected Returned Overseas Professionals in Shanxi Province [Grant Number 20210012], Project of Scientific Research for Excellent Doctors, Shanxi Province, China [Grant Number SXBYKY2021047], Research Fund (Clinical Diagnosis And Treatment Of Pet) for Young College Teachers in Ruipeng Commonweal Foundation [Grant Number RPJJ2020021], and Project of Science and Technology Innovation Fund of Shanxi Agricultural University [Grant Number 2021BQ06].

## Conflict of interest

The authors declare that the research was conducted in the absence of any commercial or financial relationships that could be construed as a potential conflict of interest.

## Publisher's note

All claims expressed in this article are solely those of the authors and do not necessarily represent those of their affiliated organizations, or those of the publisher, the editors and the reviewers. Any product that may be evaluated in this article, or claim that may be made by its manufacturer, is not guaranteed or endorsed by the publisher.
