# Supplementary material for: Pharmacokinetics and bioequivalence of two cyclosporine oral solution formulations in cats
Source: Front Vet Sci. 2022 Aug 10;9:940472. doi: 10.3389/fvets.2022.940472 (PMC9399922; doi:10.3389/fvets.2022.940472)
Supplement: Supplementary file 1 [file Data_Sheet_1.docx]

Supplementary Material

# Supplementary Data

Calibration curves showed satisfactory linearity through a concentration range of 10-2000 ng/mL (r2 ＞0.99), and the standard curve is shown in Figure 1.

Inter and intra-assay coefficients of variation were <15%. The mean recoveries ranged from 93.39 to 110.72%. (Table 1-2)

# Supplementary Figures and Tables

## Supplementary Figures

**Supplementary Figure 1.** Standard curve of cyclosporine in blood

## Supplementary Tables

**Supplementary Table 1.** Recovery of cyclosporine spiked with different batches of blood

| Drug | Concentration (ng/mL) | Day1 | Day2 | Day3 |
| --- | --- | --- | --- | --- |
| Cyclosporine | 10 | 112.05 | 98.07 | 84.49 |
|  |  | 108.43 | 88.93 | 95.57 |
|  |  | 96.94 | 116.05 | 110.27 |
|  |  | 116.61 | 109.36 | 94.85 |
|  |  | 114.81 | 106.15 | 88.88 |
|  |  | 100.76 | 114.81 | 113.67 |
|  | 20 | 84.32 | 92.76 | 114.08 |
|  |  | 110.71 | 88.9 | 114.47 |
|  |  | 109.7 | 110.25 | 104.64 |
|  |  | 111.41 | 92.75 | 113.83 |
|  |  | 107.16 | 112.31 | 111.24 |
|  |  | 89.36 | 95.9 | 106.06 |
|  | 200 | 99.14 | 88.16 | 105.72 |
|  |  | 100.79 | 85.32 | 108.29 |
|  |  | 96.03 | 92.46 | 105.68 |
|  |  | 92.78 | 97.04 | 103.12 |
|  |  | 102.99 | 101.11 | 107.89 |
|  |  | 98.14 | 96.22 | 104.82 |
|  | 1600 | 88.64 | 88.54 | 105.61 |
|  |  | 93.73 | 97.57 | 101.63 |
|  |  | 100.18 | 98.14 | 93.21 |
|  |  | 110.69 | 112.73 | 103.45 |
|  |  | 99.74 | 99.74 | 98.77 |
|  |  | 114.69 | 113.99 | 93.38 |

**Supplementary Table 2.** Recovery and coefficient of variation of cyclosporine spiked in blood

| Drug | Concentration (ng/mL) | Batch | Accuracy (%) | Intra CV(%)（n=6） | Inter CV（%）  (n=24) |
| --- | --- | --- | --- | --- | --- |
| Cyclosporine | 10 | 1 | 108.27 | 7.29 | 10.11 |
|  |  | 2 | 105.56 | 9.87 |  |
|  |  | 3 | 97.96 | 11.88 |  |
|  | 20 | 1 | 102.11 | 11.77 | 9.80 |
|  |  | 2 | 98.81 | 10.05 |  |
|  |  | 3 | 110.72 | 3.92 |  |
|  | 200 | 1 | 98.31 | 4.09 | 6.63 |
|  |  | 2 | 93.39 | 6.32 |  |
|  |  | 3 | 105.92 | 1.82 |  |
|  | 1600 | 1 | 101.28 | 9.76 | 8.06 |
|  |  | 2 | 101.79 | 9.62 |  |
|  |  | 3 | 99.34 | 5.23 |  |
